# Laboratory Test Predictors for Major Bleeding in Elderly (≥80 Years) Patients With Nonvalvular Atrial Fibrillation Treated With Edoxaban 15 mg: Sub‐Analysis of the ELDERCARE‐AF Trial

**DOI:** 10.1161/JAHA.122.024970

**Published:** 2022-09-03

**Authors:** Takeshi Mikami, Kagami Hirabayashi, Keisuke Okawa, Tetsuo Betsuyaku, Saori Watanabe, Yuki Imamura, Kimihiko Tanizawa, Takuya Hayashi, Masaharu Akao, Takeshi Yamashita, Ken Okumura

**Affiliations:** ^1^ Department of Cardiology Munakata Suikokai General Hospital Fukutsu Japan; ^2^ Department of Cardiology Tomakomai City Hospital Tomakomai Japan; ^3^ Department of Cardiology Kagawa Prefectural Central Hospital Takamatsu Japan; ^4^ Department of Rehabilitation Tokyo Tenshi Hospital Hachioji Japan; ^5^ Clinical Development Department II, Development Function, Research and Development Division Daiichi Sankyo Co., Ltd. Tokyo Japan; ^6^ Clinical Development Department III, Development Function, Research and Development Division Daiichi Sankyo Co., Ltd. Tokyo Japan; ^7^ Data Intelligence Group, Data Intelligence Department, Digital Transformation Management Division Daiichi Sankyo Co., Ltd. Tokyo Japan; ^8^ Department of Cardiology National Hospital Organization Kyoto Medical Center Kyoto Japan; ^9^ The Cardiovascular Institute Tokyo Japan; ^10^ Division of Cardiology Saiseikai Kumamoto Hospital Kumamoto Japan

**Keywords:** atrial fibrillation, creatinine clearance, edoxaban, elderly, hemoglobin, hemorrhage, prothrombin time, Arrhythmias

## Abstract

**Background:**

We investigated the predictors related to major bleeding events during treatment with edoxaban 15 mg in patients aged ≥80 years with nonvalvular atrial fibrillation and high bleeding risk, for whom standard oral anticoagulants are inappropriate, focusing on standard laboratory tests related to bleeding.

**Methods and Results:**

This was a prespecified subanalysis of the on‐treatment analysis set of the ELDERCARE‐AF (Edoxaban Low‐Dose for Elder Care Atrial Fibrillation Patients) trial. Major bleeding was the primary safety end point. The event rates were calculated according to prespecified characteristics at baseline. A total of 984 Japanese patients were randomly assigned to edoxaban 15 mg or placebo (n=492, each). During the study period, 20 and 11 major bleeding events occurred in the edoxaban and placebo groups, respectively. The adjusted analysis revealed that hemoglobin <12.3 g/dL (adjusted hazard ratio [aHR], 3.57 [95% CI, 1.10–11.55]) and prothrombin time ≥12.7 seconds; (aHR, 2.89 [95% CI, 1.05–8.02]) independently predicted major bleeding, while creatinine clearance <30 mL/min showed a tendency towards an increase in major bleeding (aHR, 2.68; 95% CI, 0.96–7.46). In patients treated with edoxaban lacking these 3 risk factors, no major bleeding occurred; major bleeding event rates increased with each risk factor. Patients with 3 risk factors were significantly more likely to have a major bleeding event at 11.05%/year (HR, 7.15 [95% CI, 1.92–26.71]).

**Conclusions:**

In elderly patients with nonvalvular atrial fibrillation with high bleeding risk, baseline hemoglobin <12.3 g/dL, prothrombin time ≥12.7 seconds, and creatinine clearance <30 mL/min may predict major bleeding during treatment with edoxaban 15 mg.

**Registration:**

URL: ELDERCARE‐AF https://www.clinicaltrials.gov; Unique number: NCT02801669.

**Nonstandard Abbreviations and Acronyms**
CrCl: creatinine clearance
NVAF: nonvalvular atrial fibrillation
OAC: oral anticoagulant

**Clinical Perspective**

**What Is New?**

- Potential risk factors for major bleeding in elderly patients with nonvalvular atrial fibrillation who are inappropriate for standard oral anticoagulants because of bleeding risks and treated with a low‐dose edoxaban were not known.
- This subanalysis of the ELDERCARE‐AF (Edoxaban Low‐Dose for Elder Care Atrial Fibrillation Patients) trial investigated the predictors related to major bleeding events during treatment with edoxaban 15 mg, focusing on the laboratory test parameters related to bleeding.
- Hemoglobin <12.3 g/dL and prothrombin time ≥12.7 seconds independently predicted major bleeding, while creatinine clearance <30 mL/min indicated a tendency towards an increase in major bleeding.

**What Are the Clinical Implications?**

- Clinicians should consider the results of laboratory tests such as hemoglobin and prothrombin time in elderly patients with nonvalvular atrial fibrillation and reduced renal function before initiating edoxaban.
- During treatment with edoxaban 15 mg, elderly patients with nonvalvular atrial fibrillation with baseline hemoglobin <12.3 g/dL, prothrombin time ≥12.7 seconds, and creatinine clearance <30 mL/min should be considered at a higher risk for major bleeding events.
- Patients with all 3 risk factors are significantly more likely to have a major bleeding event and should be monitored carefully.

Atrial fibrillation (AF) is the most common cardiac arrhythmia, with ischemic stroke being the most devastating manifestation of AF, increasing morbidity and mortality in affected patients.^1^ The use of oral anticoagulants (OAC) is recommended in clinical guidelines for the prevention of stroke, but one of the major barriers to OAC prescription is the perceived bleeding risk.^2^

Edoxaban, a direct OAC, has demonstrated noninferiority to warfarin in the prevention of stroke/systemic embolism in patients with nonvalvular AF (NVAF) and showed significantly lower rates of bleeding, even in elderly patients aged ≥75 years.^3^

However, older age is generally considered a bleeding risk and is one of the variables evaluated when assessing bleeding risk in patients with AF.^4^

The ELDERCARE‐AF trial was a randomized, placebo‐controlled trial that compared low‐dose edoxaban (15 mg once daily) with placebo in elderly (aged ≥80 years) Japanese patients with NVAF in whom the use of standard OACs (warfarin, dabigatran, rivaroxaban, apixaban, or edoxaban) was deemed inappropriate because of high bleeding risk.^5^, ^6^ Edoxaban 15 mg was superior to placebo in preventing stroke or systemic embolism, and importantly, there was no significant increase in major bleeding (hazard ratio [HR], 1.87 [95% CI, 0.90–3.89]).^5^ However, the trial showed a 1.5% absolute excess in major bleeding events (20 in the edoxaban group and 11 in the placebo group). Thus, a further assessment of the bleeding risk is required in elderly patients who may benefit from edoxaban 15 mg.^7^ As the potential risk factors for major bleeding in this elderly population with an already high bleeding risk were unknown, we conducted this subanalysis of the ELDERCARE‐AF data to investigate the predictors related to major bleeding events during treatment with edoxaban 15 mg, focusing on the laboratory test parameters related to bleeding.

## METHODS

The data sets used in the current analysis are available from the corresponding author upon reasonable request.

This was a prespecified subanalysis of data from the ELDERCARE‐AF trial, which was a phase 3, multicenter, randomized, double‐blind, placebo‐controlled, event‐driven, superiority trial. The full details of the trial design and main outcomes have been published.^5^, ^6^

ELDERCARE‐AF was conducted in compliance with Japanese legal ordinances and the ethical principles that have their origin in the Declaration of Helsinki. The protocol was approved by the ethics committee of each participating center. Written informed consent was obtained from all participants or their legal representatives before enrollment.

In brief, eligible patients were aged ≥80 years, with a documented history of NVAF within 1 year of consent and a CHADS_2_ score ≥2. In addition, patients had to be considered ineligible for treatment with standard OACs at the recommended therapeutic strength or dose because of either low creatinine clearance (CrCl; 15–30 mL/min), history of bleeding (from a critical area or organ, or gastrointestinal), low body weight (≤45 kg), continuous use of nonsteroidal anti‐inflammatory drugs, or current use of an antiplatelet drug.

Eligible patients were randomly assigned in a 1:1 ratio to receive either 15 mg of edoxaban or placebo, once daily. Randomization used an interactive response technology system and was stratified according to CHADS_2_ scores (2 or ≥3 points).

The primary safety end point was major bleeding according to the criteria of the International Society on Thrombosis and Hemostasis.^6^

The population in this subanalysis was the on‐treatment analysis set, defined as patients included in the trial treatment period and up to 3 days after the last dose of the trial drug or the end of the trial. Bleeding events were summarized by group and analyzed for the on‐treatment period. Major bleeding event rates were calculated according to prespecified characteristics determined before the administration of the trial drug. These included serum creatinine, serum alkaline phosphatase, hemoglobin, platelet count, plasma brain natriuretic peptide, serum troponin I, prothrombin time (PT), activated partial thromboplastin time, D‐dimer levels, and prothrombin fragment 1+2 levels (Data S1).

Characteristics with *P*<0.05 in the unadjusted analysis were analyzed using a Cox proportional hazard model with a single‐group indicator. CrCl was used to assess renal function instead of serum creatinine in the models because serum creatine depends on muscle mass. The CrCl <30 mL/min value was based on the severe renal impairment criteria for bleeding risk applied in this trial. CrCl (<30 mL/min), hemoglobin <12.3 g/dL (the median value), and PT ≥12.7 seconds (the median value) were included in the adjusted Cox proportional hazard model to analyze the impact on major bleeding incidence. In this trial, the median value of PT‐international normalized ratio was 1.1. The model factors were selected according to clinical expertise and statistical significance of the factors in the unadjusted analyses. Missing data were included in the analysis by imputation. No multiplicity adjustment was made. The cumulative incidence of major bleeding events was estimated by treatment group using the Kaplan–Meier method.

Statistical significance was set at *P*<0.05. Statistical analyses were conducted using SAS version 9.4 (SAS Institute Inc., Cary, NC). Takuya Hayashi had full access to all the data in the trial and takes responsibility for its integrity and the data analysis.

## RESULTS

Between August 5, 2016 and November 5, 2019, 1086 patients were enrolled, of whom 984 underwent random assignment to treatment (edoxaban 15 mg, n=492; placebo, n=492).^5^ In the placebo group, 2 patients did not receive the trial drug, so the on‐treatment population consisted of 490 patients. Out of 984 patients, 419 (42.6%) were men. No sex‐based or race‐ or ethnicity‐based differences were present (all patients were Japanese).

Twenty major bleeding events occurred in the edoxaban group and 11 events in the placebo group. Intracranial hemorrhages occurred in 2 and 4 patients, respectively, and gastrointestinal bleeding in 14 and 5 patients, respectively. There were no fatal bleeding events in the edoxaban group versus 2 in the placebo group.^5^

Table S1 shows the event rates for major bleeding events according to CrCl, hemoglobin, PT, and other laboratory parameters in the edoxaban group. The unadjusted analysis revealed that the event rate was 6.16% per year in patients with CrCl <30 mL/min (HR, 3.90 [95% CI, 1.50–10.09]), compared with those with CrCl ≥30 mL/min; 5.80% per year for hemoglobin <12.3 g/dL (HR, 4.94 [95% CI, 1.66–14.72]); and 5.05% per year for PT ≥12.7 seconds (HR, 3.13 [95% CI, 1.13–8.72]). The adjusted analysis revealed that hemoglobin <12.3 g/dL (adjusted hazard ratio [aHR], 3.57 [95% CI, 1.10–11.55]) and PT ≥12.7 seconds (aHR, 2.89 [95% CI, 1.05–8.02]) were independent predictors for major bleeding (Table 1), and CrCl <30 mL/min showed a tendency towards an increase in major bleeding (aHR, 2.68 [95% CI, 0.96–7.46]). Figure shows the cumulative incidence curves for major bleeding events according to subgroups by CrCl, hemoglobin, and PT. The placebo group showed a natural history irrespective of OAC therapy, and the event rates were listed for comparison with the edoxaban group.

**Table 1:** Baseline Characteristics and Incidence of Major Bleeding (On‐Treatment Analysis Set)

Variable	Patients (n)	Events (n)	Patient‐years	Event rate (%/y)	Unadjusted analysis		Adjusted analysis	
					HR (95% CI)	*P* value	HR (95% CI)	*P* value
Edoxaban group								
CrCl, mL/min								
<30	197	14	227.2	6.16	3.90 (1.50–10.09)	0.005	2.68 (0.96–7.46)	0.06
≥30	295	6	379.1	1.58	Reference	…	Reference	…
Hemoglobin, g/dL*								
<12.3	232	16	276.0	5.80	4.94 (1.66–14.72)	0.004	3.57 (1.10–11.55)	0.03
≥12.3	260	4	330.3	1.21	Reference	…	Reference	…
Prothrombin time, s†								
≥12.7	253	15	296.8	5.05	3.13 (1.13–8.72)	0.03	2.89 (1.05–8.02)	0.04
<12.7	239	5	309.6	1.62	Reference	…	Reference	…
Placebo group								
CrCl, mL/min								
<30	203	6	247.0	2.43	1.86 (0.57–6.05)	0.31	1.43 (0.41–4.96)	0.57
≥30	287	5	372.8	1.34	Reference	…	Reference	…
Hemoglobin, g/dL*								
<12.3	258	8	318.5	2.51	2.57 (0.69–9.54)	0.16	2.21 (0.56–8.75)	0.26
≥12.3	232	3	301.3	1.00	Reference	…	Reference	…
Prothrombin time, s†								
≥12.7	267	8	332.2	2.41	2.18 (0.55–8.65)	0.27	1.97 (0.46–8.33)	0.36
<12.7	223	3	287.6	1.04	Reference	…	Reference	…

CrCl indicates creatinine clearance; and HR, hazard ratio.

* 12.3 g/dL was the median value.

^†^ 12.7 seconds was the median value.

**Figure 1:**
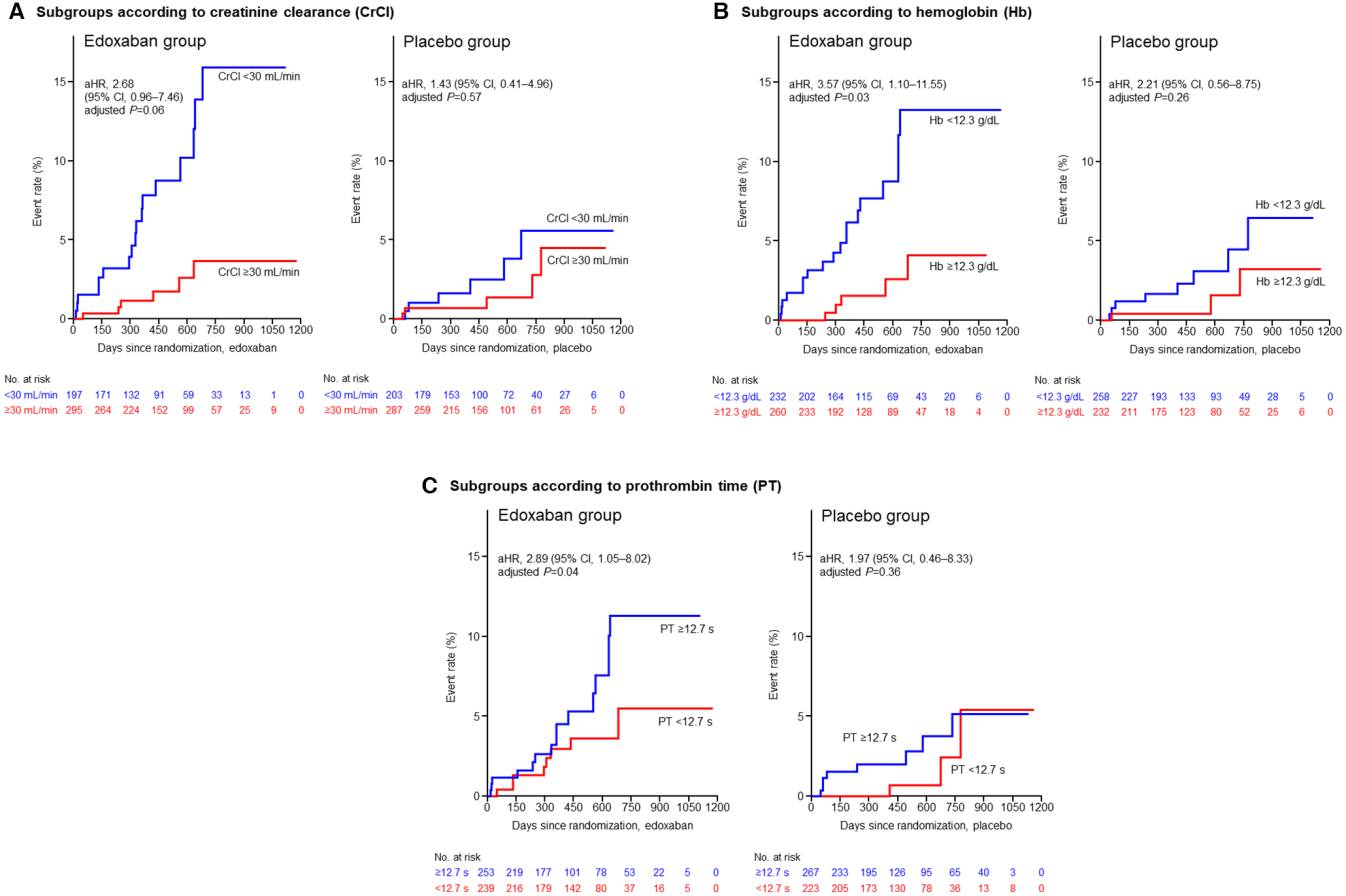
aplan–Meier curves for major bleeding events by subgroups according to (A) creatinine clearance, (B) hemoglobin, and (C) prothrombin time. Blue and red lines in each panel indicate the patient groups with and without each bleeding risk factor, respectively. aHR indicates adjusted hazard ratio; CrCl, creatinine clearance; and PT, prothrombin time.

In patients treated with edoxaban lacking any of the 3 identified risk factors (ie, CrCl <30 mL/min, hemoglobin <12.3 g/dL, and PT ≥12.7 seconds), no major bleeding occurred (Table 2). However, the major bleeding event rate increased with the increase in the risk factors, and patients with all 3 risk factors were significantly more likely to have a major bleeding event (11.05% per year; HR, 7.15 [95% CI, 1.92–26.71] versus patients with one risk factor).

**Table 2:** Incidence of Major Bleeding by Number of Bleeding Risk Factors

No. of factors*	Patients (n)	Events (n)	Patient‐years	Event rate (%/y)	Unadjusted HR (95% CI)	Unadjusted *P* value
Edoxaban group						
0	107	0	143.2	0.00	…†	…†
1	158	3	198.5	1.51	Reference	…
2	157	9	192.1	4.69	3.16 (0.85–11.78)	0.09
3	70	8	72.4	11.05	7.15 (1.92–26.71)	0.003
Placebo group						
0	90	0	116.8	0.00	…†	…†
1	161	3	213.3	1.41	Reference	…
2	150	5	184.7	2.71	2.00 (0.49–8.17)	0.34
3	89	3	105.0	2.86	2.09 (0.43–10.20)	0.36

CrCl indicates creatinine clearance; and HR, hazard ratio.

* Bleeding risk factors were CrCl <30 mL/min, hemoglobin <12.3 g/dL, and prothrombin time ≥12.7 seconds.

^†^ Not shown because interpretable estimates were not obtained due to the absence of event occurrence (HR, 0.00 [95% CI, 0.00–0.00; *P*<0.001]).

## DISCUSSION

In this post hoc subanalysis of the laboratory test factors related to major bleeding events in the ELDERCARE‐AF trial, the adjusted analysis revealed that hemoglobin <12.3 g/dL and PT ≥12.7 seconds were independent predictors for major bleeding, with CrCl <30 mL/min also showing a tendency towards an increased risk of bleeding events.

Of the many scoring systems available to predict major bleeding in patients with AF, the HAS‐BLED score remains popular because of its balance of sensitivity and specificity.^8^ Variables included in the HAS‐BLED score are hypertension, abnormal kidney/liver function, stroke, bleeding history or predisposition, labile international normalized ratio, elderly age (>65 years), and concomitant use of drugs/alcohol.^4^ The risk of major bleeding increases as the HAS‐BLED score increases (from 1.13% per year at a score of 0 to 12.50% at a score of ≥5).^4^ Our trial failed to show the increased risk of major bleeding with edoxaban 15 mg in patients with HAS‐BLED score ≥3 compared with those with the score ≤2,^5^ presumably because of the presence of bleeding risks not necessarily included in HAS‐BLED score assessments (eg, severe renal impairment defined by CrCl <30 mL/min and low body weight) in the trial. A previous study of the data derived from the ARISTOTLE (Apixaban for Reduction In Stroke and Other ThromboemboLic Events in Atrial Fibrillation) trial proposed the ABC‐bleeding score consisting of age, biomarkers (growth differentiation factor‐15, high‐sensitivity cardiac troponin T, and hemoglobin), and clinical history of previous bleeding, which yielded a higher c‐index than HAS‐BLED and Outcomes Registry for Better Informed Treatment (ORBIT) scores for major bleeding.^9^ Furthermore, a modified ABC‐bleeding score using an alternative biomarker CrCl also outperformed HAS‐BLED and ORBIT scores.

CrCl <30 mL/min is an indicator of severely impaired kidney function, hemoglobin <12.3 g/dL indicates a predisposition to bleeding, and all patients in the ELDERCARE‐AF trial were elderly (≥80 years). Because the labile international normalized ratio is a factor specific to patients taking warfarin, we consider that a median PT ≥12.7 seconds is more applicable to patients taking direct OACs. Thus, the 3 risk factors in our trial (CrCl <30 mL/min, hemoglobin <12.3 g/dL, and PT ≥12.7 seconds) may represent the risk for major bleeding in elderly patients. Like HAS‐BLED, bleeding rates in our analysis increased as the number of risk factors increased, from 0% per year for edoxaban‐treated patients with no risk factors to 11.05% per year for those with 3 risk factors.

Of note, the 3 risk factors identified in our subanalysis are consistent with the published literature. Multiple previous studies have explored the link between impairment in kidney function and AF, and it is well known that chronic kidney disease is associated with an increased risk of stroke or systemic thromboembolism and bleeding among patients with AF.^10^ Moreover, data from the Fushimi AF Registry of Japanese patients with AF stratified by CrCl showed that after adjustment for prespecified factors, patients with CrCl <30 mL/min had an increased risk of both stroke/systemic embolism (HR, 1.68 [95% CI, 1.04–2.65; *P*=0.04]) and major bleeding (HR, 2.08 [95% CI, 1.23–3.39; *P*=0.008]).^11^ Renal function is an important determinant of plasma edoxaban concentration and the decline in the function is likely to be associated with the increased drug concentration. We consider a periodic examination renal function (creatinine clearance) is mandatory in elderly AF patients at risk of renal impairment as indicated by the European Heart Rhythm Association practical guide.^12^

Anemia is a frequently observed comorbidity in patients with AF and has been reported to be associated with an increased risk of major bleeding, as well as with all‐cause mortality, cardiovascular and noncardiovascular mortality, stroke/systemic thromboembolism, and gastrointestinal bleeding.^13^ However, we note that patients with hemoglobin <9 g/dL were excluded from this trial, which may have introduced some bias, and physicians will likely consider hemoglobin <12.3 g/dL as marginally low in the general population. However, this criterion seems to be a risk factor of major bleeding in this population of elderly patients.

Although there are no data indicating that baseline PT before the administration of OACs is a predictor of major bleeding, it has been shown that bleeding event rates were higher in patients treated with direct OAC with excessive prolongation of coagulation attributable to inappropriately high dosing and/or low body weight.^14^ It is known that PT can be prolonged as plasma edoxaban concentrations increase and that the area under the blood concentration–time curve is increased when edoxaban is administered to patients with renal dysfunction (which can include elderly patients with age‐related kidney impairment).^15^ Thus, if baseline PT is long, the effect of edoxaban is likely to be strong, and the risk of bleeding will be higher. It may be argued that a PT of <13.5 seconds is normal, based on some laboratory findings in general populations. However, the present trial of elderly patients with NVAF and high bleeding risk at baseline showed that increases in PT ≥12.7 seconds at baseline may predict major bleeding in this population. The median value was used to reduce the bias that may have arisen from the difference in the number of patients with and without risk.

A prespecified subanalysis of the ENGAGE AF‐TIMI 48 (Effective Anticoagulation with Factor Xa Next Generation in Atrial Fibrillation–Thrombolysis in Myocardial Infarction 48) trial evaluated clinical outcomes of edoxaban versus warfarin in patients with AF according to age. Elderly patients (aged ≥75 years) treated with edoxaban were shown to derive therapeutic benefit.^3^ On the other hand, the risk of major bleeding exceeded the risk of stroke or systemic embolic events with increasing edoxaban concentration, resulting in a narrow therapeutic window.^16^ As such, while the results of the ELDERCARE‐AF trial suggest that prescribing low‐dose edoxaban (15 mg/day) can prevent stroke or systemic embolism in elderly patients with AF in whom other OACs are unsuitable,^5^ it remains important to consider the risk of bleeding. Thus, the findings of this subanalysis suggest that clinicians should particularly consider the results of laboratory tests such as hemoglobin and PT in elderly patients with reduced renal function before initiating edoxaban.

The present trial has some limitations. The restricted patient numbers (492 edoxaban‐treated individuals from Japan) may limit the generalizability. The small number of bleeding events may have confounded the data, particularly as there were no events in patients lacking all 3 identified risk factors. In the present analysis, we focused on the routine laboratory tests relating to the increased risk of major bleeding, and the concentration of edoxaban was not assessed. Its relation to the efficacy and safety seen in the ELDERCARE‐AF trial seems to be important, and a further study would be required. We used the result of PT test as a bleeding risk factor, instead of its normalized ratio, PT‐international normalized ratio. In this trial, the result of PT test was reported in 3 significant digits whereas PT‐international normalized ratio in 2 significant digits. Therefore, the result of PT was preferable to evaluate the risk of bleeding events more sensitively. This analysis of predictors was based on baseline laboratory test values (such as kidney function) that may have changed during the trial period. A large proportion of patients discontinued the ELDERCARE‐AF trial because of their high‐risk status. However, no patients were lost to follow‐up, and only 6 withdrew consent because of bleeding‐related concerns. Most of those who withdrew did so because of adverse events unrelated to bleeding or because they were no longer capable of participation. A separate analysis was conducted to evaluate whether patient discontinuation influenced the results; however, similar results to those of the primary analysis were obtained. This trial involved Japanese patients with AF, and therefore the results may not apply to other ethnic populations. The trial sample size was not powered for any subgroup analyses, and the event rates in the subgroups were low. Finally, other factors such as age, sex, body mass index, or comorbidities were not considered in this subanalysis.

## CONCLUSIONS

In elderly patients with NVAF, for whom standard OACs are inappropriate because of high bleeding risk, baseline hemoglobin <12.3 g/dL, PT ≥12.7 seconds, and CrCl <30 mL/min may be predictive for major bleeding during treatment with edoxaban 15 mg.

## Sources of Funding

This trial was supported by Daiichi Sankyo Co., Ltd.

## Disclosures

Akao received funding support for the present work from Daiichi Sankyo Co., Ltd., as well as grants or contracts from Bayer Yakuhin, Ltd., and payment and honoraria from Pfizer Japan Inc., Bristol‐Myers Squibb K.K., Nippon Boehringer Ingelheim Co., Ltd, Bayer Yakuhin, Ltd., and Daiichi Sankyo Co., Ltd. outside the submitted work. Betsuyaku received payment and honoraria from Novartis Pharma K.K. and Daiichi Sankyo Co., Ltd. outside the submitted work, and owns stock or stock options from Daiichi Sankyo Co., Ltd. and Takeda Pharmaceutical, Inc. Hirabayashi received payment and honoraria from Daiichi Sankyo Co., Ltd., Novartis Pharma K.K., Ono Pharmaceutical Co., Ltd, AstraZeneca K.K., and Bayer Yakuhin, Ltd. outside the submitted work. Hayashi, Imamura, Watanabe, and Tanizawa are employees of Daiichi Sankyo Co., Ltd. Mikami and Okawa have no conflicts of interest to declare. Yamashita received personal fees from Daiichi Sankyo Co., Ltd. during the conduct of the study; grants and personal fees from Daiichi Sankyo Co., Ltd., Bayer Yakuhin, Ltd., and Bristol Myers Squibb K.K.; personal fees from Pfizer Japan Inc., Nippon Boehringer Ingelheim Co., Ltd., Ono Pharmaceutical Co., Ltd., Toa Eiyo Ltd., and Novartis Pharma K.K. outside the submitted work. Okumura received grants and personal fees from Daiichi Sankyo Co., Ltd. during the conduct of the study; personal fees from Daiichi Sankyo Co., Ltd., Nippon Boehringer Ingelheim Co., Ltd., Bristol‐Myers Squibb K.K., Medtronic Japan Co., Ltd., Johnson & Johnson K.K., and Bayer Yakuhin, Ltd. outside the submitted work.

## Supporting information

Data S1
Table S1
Reference 17
