# Supplementary material for: Laboratory Test Predictors for Major Bleeding in Elderly (≥80 Years) Patients With Nonvalvular Atrial Fibrillation Treated With Edoxaban 15 mg: Sub‐Analysis of the ELDERCARE‐AF Trial
Source: J Am Heart Assoc. 2022 Sep 3;11(17):e024970. doi: 10.1161/JAHA.122.024970 (PMC9496443; doi:10.1161/JAHA.122.024970)
Supplement: Supplementary file 1 — Data S1 Table S1 Reference 17 [file JAH3-11-e024970-s001.pdf]

SUPPLEMENTAL MATERIAL

Data S1. Supplemental Methods

All laboratory tests were performed before the administration of the trial drug at a central laboratory (SRL, Inc., Tokyo, and Kanagawa, Japan).

Rationale for the cutoff points

The cutoff point for CrCl is 30 mL/min as this was defined as being at risk of bleeding in the inclusion criteria.

The cutoff point for BNP is 200 pg/mL based on the statement from the Japanese Heart Failure Society that suggests a cutoff point of 200 pg/mL as “highly likely to have HF which requires treatment, and extensive testing or referral to a specialist should be conducted.”[17]

The other cutoff points were medians (hemoglobin, troponin I, prothrombin time, activated partial thromboplastin time, D-dimer, and prothrombin fragment 1+2 levels) or the nearest integer point to the median (alkaline phosphatase: median, 249.0 U/L; platelets: median, 19.9×10^4/μL).

As a sensitivity analysis, we identified the optimal cutoff values for the major bleeding event based on Youden Index: hemoglobin: 12.0 g/dL, prothrombin time: 12.5 sec. These optimal cutoff values are close to their medians respectively (median: hemoglobin: 12.3 g/dL, prothrombin time: 12.7 sec.). Therefore, we consider the present categorization by median values was reasonable.

Laboratory data collection methods

Laboratory data were collected using the following methods: serum creatinine[a] (measured with enzyme method; Hitachi 7700 Series), serum alkaline phosphatase[a] (JSCC standardized method; Hitachi 7700 Series), hemoglobin[b] (sodium lauryl sulfate-hemoglobin method; Sysmex Automated Hematology Analyzer XE-2100), platelet count[b] (Hydro Dynamic Focusing, DC Detection; Sysmex Automated Hematology Analyzer XE-2100), plasma brain natriuretic peptide[c] (chemiluminescence enzyme immunoassay; Lumipulse Presto II (applicable for samples accepted up to 28 Feb 2017) or Lumipulse L2400 (applicable for samples accepted from 29 February 2017), serum troponin I[d] (chemiluminescence enzyme immunoassay; UniCel Dxl 800), prothrombin time[e] (blood clotting time measurement method; Sysmex CS-2400 Series), activated partial thromboplastin time[e] (blood clotting time measurement method; Sysmex CS-2400 Series), D-dimer levels[e] (latex immunoassay; JCA-BM1650 (applicable for samples accepted up to 6 Sep 2018) or JCA-BM8020 (applicable for samples accepted from 7 Sep 2018)), and prothrombin fragment 1+2 levels[e] (enzyme-linked immunosorbent assay; EMax Microplate Reader).

[a]Blood was collected using vacuum blood sampling tubes. The serum was stored refrigerated.

[b]Blood was collected using vacuum blood sampling tubes containing EDTA-2K and stored refrigerated.

[c]Blood was collected using vacuum blood sampling tubes containing EDTA-2Na. The plasma was stored frozen.

[d]Blood was collected using vacuum blood sampling tubes. The serum was stored frozen.

[e]Blood was collected using vacuum blood sampling tubes with sodium citrate. The plasma was stored frozen.

Table S1. Event Rates for Major Bleeding Events According to Laboratory Test Parameters Related to Bleeding in the Edoxaban Group

Variable	Patients (N)	Events (n)	Patient-years	Event rate (%/y)	Unadjusted HR (95% CI)	Unadjusted *P* value
CrCl (mL/min)						
<30	197	14	227.2	6.16	3.90 (1.50–10.09)	0.005
≥30	295	6	379.1	1.58	Reference	-
Alkaline phosphatase (U/L)						
≥250	241	13	290.1	4.48	1.99 (0.79–5.00)	0.14
<250	251	7	316.2	2.21	Reference	-
Hemoglobin (g/dL)						
<12.3 (median)	232	16	276.0	5.80	4.94 (1.66–14.72)	0.004
≥12.3 (median)	260	4	330.3	1.21	Reference	-
Platelets (10^4/µL)						
<20	244	13	292.2	4.45	2.02 (0.80–5.05)	0.13
≥20	248	7	314.1	2.23	Reference	-

Plasma BNP (pg/mL)						
≥200	270	14	321.7	4.35	2.04 (0.78–5.34)	0.15
<200	222	6	284.6	2.11	Reference	-
Troponin I (ng/mL)						
≥0.02 (median)	246	13	297.4	4.37	1.89 (0.76–4.69)	0.17
<0.02 (median)	246	7	308.9	2.27	Reference	-
Prothrombin time (s)						
≥12.7 (median)	253	15	296.8	5.05	3.13 (1.13–8.72)	0.03
<12.7 (median)	239	5	309.6	1.62	Reference	-
Activated prothrombin time (s)						
≥27.3 (median)	245	12	278.9	4.30	1.75 (0.71–4.33)	0.23
<27.3 (median)	247	8	327.4	2.44	Reference	-
D-dimer (µg/mL)						
≥1.15 (median)	236	9	268.0	3.36	1.04 (0.44–2.48)	0.93
<1.15 (median)	252	11	332.7	3.31	Reference	-
Missing	4	0	5.6	0.00	-*	-*

Prothrombin F1+2 (pmol/L)						
<421 (median)	245	11	306.8	3.59	1.16 (0.48–2.78)	0.74
≥421 (median)	247	9	299.5	3.00	Reference	–

BNP indicates brain natriuretic peptide; CI, confidence intervals; CrCl, creatinine clearance; and HR, hazard ratio.

*Not displayed in this table because interpretable estimates were not obtained due to the absence of event occurrence.

(HR, 0.00 [95% CI, 0.00–0.00; *P*<0.001]).
